# Brain and Subjective Responses to Indoor Environments Related to Concentration and Creativity

**DOI:** 10.3390/s24237838

**Published:** 2024-12-08

**Authors:** Ze-Yu Wang, Ji Young Cho, Yi-Kyung Hong

**Affiliations:** Department of Housing & Interior Design (AgeTech-Service Convergence Major), Kyung Hee University, Seoul 02447, Republic of Korea; taeku93@khu.ac.kr (Z.-Y.W.); lucis@khu.ac.kr (Y.-K.H.)

**Keywords:** brain response, concentration index, indoor environment, relative theta, subjective response

## Abstract

Electroencephalograms (EEGs) can be used to study the influence of environmental elements on human emotions, cognition, and behavior. EEGs can reveal unconscious responses and fill in the gaps left by subjective responses provided in survey questionnaires or interviews. EEG research on the impact of classroom design elements on concentration and creativity is scarce; the design elements studied have not been diverse enough. In addition, no researchers have examined the brain and subjective responses to multiple indoor environmental elements regarding concentration and creativity. Thus, the purpose of this study was to explore how the human brain responds to different indoor environmental elements as shown by objective EEG signals related to concentration and creativity, and their similarities and differences to subjective self-reported responses. The experimental stimuli included 16 images combining four indoor environmental elements—classroom space shape, furniture arrangement, ceiling height, and color—along with images of white walls, a full-window wall with a view of nature, and a windowless scenario, totaling 19 images. The brainwaves of 20 people collected from eight channels were analyzed to determine the concentration index (CI) for concentration and relative theta (RT) for creativity. As a subjective response, participants were asked to choose the stimuli in which they felt they could best concentrate and be most creative in a self-report format. The results showed the following tendencies: (a) More brainwaves in the parietal and occipital lobes than in the prefrontal or frontal lobes; (b) a higher CI with rectilinear shapes, traditional frontal furniture arrangements, and red walls; (c) a higher RT with curvilinear shapes, collaborative furniture arrangements, white walls, and a full view of nature; and (d) participants selected white walls and a front-facing furniture layout as supportive of concentration and a full view of nature, curvilinear shape, and collaborative furniture layout for creative thinking. The results showed that similarities in brain and subjective responses were related to furniture layout and shape, whereas differences existed in color. This study contributes to the understanding of the characteristics of indoor environments that appear to enhance the manifestation of concentration and creativity.

## 1. Introduction

Elements of the indoor environment, such as color, layout, lighting, scent, and sound, can significantly influence human emotions and behavior [1]. These effects can be identified through various methods, including questionnaires, facial expression analysis, eye-tracking, and electroencephalography (EEG) [2,3]. Brain sensors are used in electroencephalograms (EEGs) to understand brain responses noninvasively. The strengths of EEG include affordability, cost-effectiveness, portability, and high temporal resolution [4,5] that can indicate cognitive activities and mental states. Although brainwaves do not directly indicate the mental status of the observed person, they can allow inferences about them, and the reasons for these responses can be inferred by synchronizing the stimuli and brain response. Brainwave responses may reveal unintentional, unconscious responses which differ from intentional responses that may appear in subjective inquiries like surveys or interviews [6].

Classroom environments in which students and teachers engage are significant in student learning. Physical classroom environments exert cognitive, emotional, and physiological effects [7]. Subjective surveys have been the primary means of understanding how people feel about environmental design. However, the recent availability of affordable EEG equipment has led to some publication of studies on understanding brain responses to classroom environments through objective physiological measurements [8,9]. Without changing the entire structure of the spaces, modification of spatial elements, including shape, furniture arrangement, and color, may allow changes that result in substantial differences. Moreover, identifying such effects may help designers and educators make decisions regarding the development of prototypes in future classrooms.

Concentration and creativity are essential in solving problems. Concentration is the ability to focus on a specific task or stimulus despite various distractions and sustain attention for a continuous period [10]. Although individual differences may exist, environmental characteristics can diversely affect concentrations. For instance, excessive noise, extreme darkness or light, and uncomfortable temperature and humidity can hinder concentration [11]. Creativity, the ability to solve problems and produce original, useful results [12], helps connect known information in new ways to generate and develop ideas [13], especially connecting relatively remote concepts or ideas [14]. Enhancing creativity is considered an important goal of education [15]. Without the manifestation of creativity in daily life, innovative problem-solving is difficult, if not impossible; therefore, an educational environment fostering the expression and development of creativity is essential.

In this context, examining the environmental characteristics of educational spaces that facilitate concentration and creativity, particularly the classroom environment, seems important. After the COVID-19 pandemic, students’ decreased attention, reduced cooperative consciousness, and passive learning attitudes have raised concerns. According to the 2022 Annual Reports published by the Center for Collegiate Mental Health [16], 61.1% of 123,865 students who received counseling at American colleges reported that the pandemic had a negative impact on their motivation or focus.

Recently, studies have been conducted to identify indoor environmental characteristics related to concentration and creativity in educational spaces using EEG [17,18,19], but neurophysiological evidence using EEG has not been extensively explored [11]. EEG research on the impact of classroom design elements on concentration and creativity is scarce; the design elements under investigation have not been sufficiently diverse. Furthermore, no studies have examined brain and subjective responses to elements of multiple indoor environments regarding concentration and creativity; thus, further research is necessary.

The purpose of this study was, therefore, to understand (a) how the human brain responds to various indoor environmental elements—space shape, furniture arrangement, ceiling height, color, and view of nature—as shown by objective EEG signals related to concentration and creativity, and (b) how the responses are similar or different from subjective self-reported responses, aiming to determine which elements best enhance spaces conducive to concentration and creativity. Identifying such elements will be helpful for the design of classroom environments, as appropriate environmental settings can help individuals and communities perform creatively and cognitively well [20]. As brain response indicators, based on a literature review, the concentration index (CI) was used because it has been used to measure concentration [21,22,23], and the relative theta (RT) because of its potential correlation with creativity [24,25].

The research questions for this study are as follows:

1. How do brainwave responses associated with concentration and creativity vary with changes in interior design elements (shape, furniture arrangement, ceiling height, color, and view of nature)?
2. How do they differ by EEG channel?
3. For which stimuli are the brainwaves significantly different from those of the prestimulation?
4. Overall, which interior design elements tend to activate brainwaves relevant to concentration and creativity?
5. According to participants’ subjective responses, which stimuli do they believe will help them focus, and which will help them think creatively?
6. How similar or different are the brain’s responses and the subjective responses?

## 2. Literature Review

### 2.1. Spatial Elements Affecting Concentration and Creativity

One of the most important sensory channels for human beings is vision, and in any environment, shape, size, and color constitute the main visual dimensions [26]. Among the elements that organize a classroom environment, the key components include the shape of the space, arrangement of furniture, height of the ceiling, and color of the walls.

#### 2.1.1. Shape

The space shape comprises the configuration of the interior environment, the outline of the room, and the contours of elements in the room. Shapes are diverse and classified into geometric, organic, and free forms; however, they can be divided into two categories: rectilinear (linear) and curvilinear (curved). Research on the relationship between the shape of space and concentration is limited, so recognizing that concentration is influenced by various factors beyond space shape is important. The only cue that could be obtained was from the inferences of two separate studies. For example, (a) highly arousing affective states lead to higher attention [27], and (b) spaces rated with higher pleasure and arousal ratings often incorporate curved geometries, while those with lower pleasure and arousal ratings are more likely to include linear geometries [17]. Banaei et al. (2017) revealed the effect of the shape of space on human brain activity through EEG, showing that curves have a strong effect on theta wave activity in the anterior cingulate cortex (ACC), meaning that curved spaces or structures trigger higher emotional activation [17]. Nevertheless, other researchers have reported no relationship between form and arousal [28].

Regarding shape and creativity, curvilinear shapes have been reported to relate to creativity. For example, a physical work environment with rounded furniture and equipment was reported to enhance divergent thinking, one of the characteristics of creative thinking, while angular shapes enhanced convergent thinking [29]. Curvilinear forms evoke more pleasant emotions than rectilinear forms [30], and people solve creative problems more effectively when in a pleasant mood [31]. Thus, we infer that curvilinear shapes will likely facilitate greater creativity than rectilinear shapes.

#### 2.1.2. Furniture Arrangement

The table arrangement in a classroom can have a positive or negative impact on learning. Traditional linear table arrangements that face the front tend to help maintain students’ attention to the teacher but often limit interaction and collaboration among students. Conversely, table configurations for collaboration, in which tables are organized into groups, encourage face-to-face interactions among students, allow for easy movement [32], and potentially benefit creativity, but may interfere with focus on the teacher. In a study of physical spaces with open-ended questions about innovation, five characteristics of innovative spaces were identified: collaboration and communication-enabling, modifiable, intellectual, attracting, and value-reflecting spaces [33]. The purpose of such innovative spaces was to promote creativity. Flexible and diverse table arrangements have also been demonstrated to enhance creativity in discussion-based lectures [34].

#### 2.1.3. Ceiling Height

Empirical research on the relationships among ceiling height, concentration, and creativity is scarce [12]. Meyers-Levy and Zhu [35] investigated responses in rooms with 8-foot and 10-foot ceilings. They found that people in rooms with 10-foot ceilings tended to think more freely and abstractly and could solve more than twice as many creative problems as those in rooms with lower ceilings, whereas those in rooms with 8-foot ceilings were more likely to focus on details. Their work suggests that lower ceilings are likely associated with concentration, while higher ceilings are correlated with free and abstract thinking and, potentially, creativity.

Zhang et al. (2024) investigated the effects of classroom size and ceiling height on learning outcomes using virtual reality technology [18]. They found significant differences in task test scores for different ceiling heights in classrooms of the same size. Participants in the SLR (Small and Low Room: 9.0 × 4.5 × 3.0 m) classroom had the highest scores and performed better on the tests, especially in cognitive reactivity and logical thinking. Furthermore, the physiological data, EEG-β power, increased by 53.9% in the BHR (Big and High Room: 9.0 m × 6.9 × 4.8 m) compared to the BLR (Big and Low Room: 9.0 × 6.9 × 3.9 m); in the SLR, it increased by 22.8% compared to the SHR (Small and High Room: 9. 0 × 4.5 × 3.9 m). In other words, beta power was higher for higher ceilings in larger classrooms and lower ceilings in smaller classrooms, suggesting that ceiling height, in conjunction with classroom size, affects learning performance.

#### 2.1.4. Color

Prior studies on colors that enhance concentration have shown mixed results. Red color has been reported to improve performance in tasks requiring attention to detail, suggesting that concentration may be better in red environments [36]. However, contrasting results have been reported, showing that the effect of concentration (concentration index in EEG) was lower in red environments than in green environments [19]. The heart rates of participants were consistently lower in red rooms than in blue ones; moreover, in a red environment, accuracy in proofreading and correcting tasks decreased, especially for those in a negative mood [37]. The concentration was highest in purple environments, followed by blue, green, yellow, and red [8]. The relationship between concentration and color remains unclear, as pointed out in a recent review paper [38].

Regarding color and creativity, prior studies have indicated that cool colors, particularly blue, facilitate creativity [39]. Blue and red accent lighting were found to indirectly improve creative performance by providing motivation during task completion and answering questions [40]. Blue was also found to initiate approach motivation and encourage the acceptance of new ideas; furthermore, it enhanced performance in creative tasks [36].

#### 2.1.5. View of Nature (Biophilia)

Previous studies have demonstrated that nature improves concentration and creativity. College students performed better when they were in a classroom with a natural view than in a classroom with a concrete retaining wall [41], and contact with nature brought benefits in regaining concentration from mental fatigue [42]. Additionally, the experience of garden scenes in VR has been shown to increase individual creativity [43]. In a study on the influence of virtual natural environments on creativity during product design activities, individuals tended to be more creative in natural environments than in settings without plants [44]. The multisensory stimulation of natural environments was reported to help in creative actions and thinking [45]. Similarly, natural elements are beneficial for concentration and creativity.

### 2.2. EEG and Brainwaves

#### 2.2.1. EEG, Brainwaves, and the Brain

Electroencephalography refers to the measurement of electrical activity in the brain arising from ion flow within neurons [46]. This complex brainwave signal can be recorded using EEG devices, and diverse frequencies of brainwaves can be obtained through Fourier transform. Brainwaves are divided into wavelengths based on their frequency domains. In the order of low to high frequencies and slow to fast waves, brainwaves are categorized as delta waves (0–4 Hz), theta waves (4–8 Hz), alpha waves (8–13 Hz), beta waves (13–30 Hz), and gamma waves (>30 Hz) [47]. Low-frequency waves indicate that the brain is not considerably active and usually appear when the brain is in processing. Delta waves, the slowest, are active in deep sleep, while theta waves appear in light sleep during meditation and creative thinking. Alpha waves tend to appear during relaxation, meaning calm emotion; beta waves appear during concentration, tension, arousal, and alertness, and gamma waves appear in high-level cognition or excitement [46].

The cerebral cortex functions as a unified whole in complex cognitive processes; it is divided into distinct regions responsible for specific functions. For example, the prefrontal cortex is primarily involved in higher cognitive functions, the frontal lobe in cognition and thinking, the parietal lobe in integrating external information, action, and spatial information, the temporal lobe in auditory and speech information, and the occipital lobe in visual activities [48,49].

#### 2.2.2. EEG Indicators Related to Concentration and Creativity

Researchers have developed a metric called the concentration index (CI) as an indicator of concentration, calculated as $\frac{sensorimotor\;rhythm\left(SMR\right)+mid\;beta(m\beta )}{theta(\theta )}$, with higher values indicating greater attention [49,50,51]. In this formula, SMR falls in the frequency range of 12–15 Hz, and mid-beta falls in the range of 15–20 Hz. In a study about low-arousal hyperkinesis in children, Lubar and Lubar (1984) [52] found that when theta increased, SMR or mid-beta decreased; however, improvements in school performance typically followed an increase in SMR or mβ. The SMR wave is known to be dominant during relatively simple tasks, whereas mid-beta is dominant during the performance of activities requiring a mental load, such as calculations and mental arithmetic [50,53]. Elevated mid-beta values are known to associate with increased attention and focused states and are present during tasks that require mental effort, such as work and learning [50]. CI has been used in prior studies on concentration [48,49,50], and a positive correlation between CI and cognitive test performance has been reported [50]. Therefore, we used the CI as the concentration index.

Identifying a specific brainwave indicator of creativity is challenging; related research is scarce because of the complex and multifaceted nature of creativity, which varies in its domain (i.e., general creativity vs. domain-specific creativity) and characteristics (divergent creativity vs. convergent creativity). Although few studies have been conducted, some researchers have explored the relationship between theta waves and creativity. Theta waves are present in various parts of the brain and are involved in cognitive and noncognitive processes, attention, and sleep [46,48]. Theta waves are known to exhibit abrupt changes at key moments of insight during synthesis thinking, when individuals integrate newly acquired information in a creative way to solve complex problems. This shift in brainwave patterns reflects the process of creative problem-solving and can indicate moments when difficult technical concepts suddenly make sense [54]. Theta waves are also associated with memory, which suggests that combining old and new thoughts can lead to creative outcomes. In research related to human working memory, the peak frequencies of theta and alpha waves are associated with the capacity of human working memory, particularly in the frontal lobe region [24]. Additionally, in a study on creative potential and EEG, participants who performed well in a creativity task (the compound remote associates task) showed increased theta and alpha wave activity over the left temporal lobe [25], indicating a potential correlation between theta waves and creativity. Therefore, we used relative theta (RT) as an indicator of creativity.

#### 2.2.3. EEG-Based Analysis of Classroom Design for Concentration and Creativity

We examined existing studies in which EEG on the impact of classroom environment design related to concentration and creativity. Because the papers from the perspective of creativity were not related to the physical environment, a total of 11 studies related to concentration were reviewed. The main findings are presented in Table 1.

Of the eleven studies, one dealt with the height of the space [18], two with color [8,9], two with natural or biophilic design [55,56], three with lighting [57,58,59], one with the width of the space [60], and one with the lighting, sound, temperature, and smell of the space [61]. In the remaining study, real and virtual environments were compared [62]. Regarding color, a virtual classroom with warm-colored walls (yellow) induces higher low-β and high-β frequencies in the FP2 channel and better learning outcomes than a classroom with white walls [9]. Moreover, one study reported that cold hues induce higher beta waves and better attention and memory task performance than warm colors [8]. A significant difference in task scores was reported depending on the ceiling height in classrooms of the same size [18]. Windows with a view of natural scenery resulted in better learning outcomes compared to architectural windows [56], and classrooms with natural elements incorporated into the walls and floors showed improved learning performance [55]. Research has indicated that concentration levels are similar in virtual and real environments [62].

Overall, we found a limited number of empirical studies and mixed results in the findings regarding brain responses to the classroom environment using EEG. Several researchers have reported conflicting results regarding the impact of classroom environment design on EEG responses. For example, Llinares et al. (2021) found that cool hues enhance arousal levels and improve performance in attention and memory tasks [8], but Liu et al. (2022) reported that warm-colored walls (yellow and red) yield better attention and learning outcomes [9]. This emphasizes the need for a deeper understanding of how physical environmental factors impact human response and highlights the necessity for further research on the interactions among various environmental factors. In summary, EEG research on the impact of classroom design elements on concentration and creativity is scarce; the design elements under investigation were not sufficiently diverse. The components of a classroom environment encompass more than light, color, room width, and biophilia. These can include the shape of the space, furniture arrangement, ceiling height, and view of nature, among others; and thus, the need to expand research to include such elements is clear. Therefore, this study focused on examining four components of classroom environments that could potentially enhance concentration and creativity: space shape, furniture arrangement, ceiling height, and color.

## 3. Research Methods

### 3.1. Participants

This study was approved by the Institutional Review Board (IRB) of Kyung Hee University. The experiment was conducted from May 30 to June 11, 2023. Participants were recruited through an announcement sent to people on the mailing list of the college to which the researchers were affiliated at Kyung Hee University, and the applications were accepted. A total of 21 participants without visual or color perception problems (e.g., color blindness or color weakness) were selected from among undergraduates, graduate students, and staff at Kyung Hee University. One participant was excluded because they did not meet the age criteria, leaving 20 participants, including 6 males (33%) and 14 females (67%), with an age range of 18–29 years and an average age of 22.7 years. Among them, twelve majored in housing design-related fields, and eight pursued other majors.

### 3.2. Experimental Stimuli

The experimental stimuli were designed based on a typical classroom environment. Developed using SketchUp 2019 (Trimble Inc., Sunnyvale, CA, USA) and Enscape 3.3.0 (Enscape GmbH, Karlsruhe, Germany), the classroom environment stimulus was made at a size of 14 × 8.3 m. The size and height of the classrooms at the university to which the researchers were affiliated were used as a reference to set up a typical school classroom with furniture arranged in a layout accommodating tables and chairs. The elements of this stimulus included space shape (curvilinear vs. rectilinear), furniture arrangement (collaborative vs. traditional), ceiling height (4 vs. 2.7 m), and color (blue vs. red), combined into 16 different sets. The space shape involved the following elements: the walls of the classroom space, the shape of ceiling lighting, ceiling lighting arrangement, and corner details of the tables. Regarding the furniture arrangement, based on research findings [32], we set up a traditional desk layout with four people sitting side-by-side facing the front of the room and a collaborative layout with six people sitting in clusters facing each other. Each space was designed with 48 seats. The ceiling heights were set at 2.7 m for the lower and 4 m for the higher, almost 1.5 times higher than the lower, to create a noticeable difference in the perception of ceiling height. The selected colors were deep blue and deep red from the hue and tone system presented by Korea’s Image Research Institute (IRI). The colors were 8.7B 4/5 and 2.5R 4/8 on the Munsell Color Palette. The IRI color system is a standard color system with reliable validity for Korean emotions, as used in other relevant studies [19,63]. We chose these colors based on a study by Sea et al. (2021), who found that the color with the lowest concentration was red-deep and that there was a correlation between color images and attention [19]. We also chose blue based on a study by Xia et al. (2016), which found that blue is associated with creativity [64] and chose deep blue with the same hue as deep red to match the hue of these two colors. Three types of contrasting walls were added: a common white wall, usually seen in typical classrooms; a wall made entirely of glass, revealing the natural scenery outside; and a blue wall without windows. A total of 19 stimuli were generated (Table 2).

### 3.3. Experimental Environment and Procedure

The experiment was designed to measure the brainwaves emitted when viewing each classroom image in a natural state based on other EEG studies [19,55]. Administering a different concentration and creativity test for each image would have been ideal to compare results, but doing so with a total of 19 stimuli was not appropriate as it would have been cognitively demanding for participants. Therefore, we planned to use the first experiment to identify key stimuli, then compare EEG, subjective, and task performance with fewer impactful stimuli in subsequent studies.

**Figure 1:**
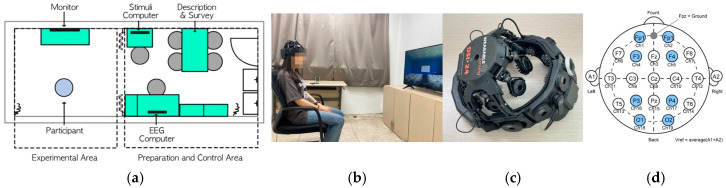
(**a**) Experimental lab floor plan; (**b**) participant in front of a monitor; (**c**) dry EEG equipment (DSI-24); (**d**) eight channel sensor locations.

The experiment was conducted at Kyung Hee University in a laboratory, the dimensions of which were 8.24 × 2.88 m with a ceiling height of 2.73 m. The area was divided into an experimental area and a preparation area using sliding doors. Soundproofing materials were installed to block external noise on window sides and at the entrance door, and the indoor temperature and humidity were set to approximately 23 °C and 66%, respectively. The laboratory ceiling had two 50 w (5700 K) LED light sources, each measuring 1.28 m × 0.32 m; the illuminance at the participant’s seat was set to 800lux. A 43-inch monitor was installed in the experimental area at a height appropriate for the participant’s eye level (Figure 1a,b).

The EEG device used was a Wearable Sensing DSI-24 Dry EEG system (Figure 1c), and measurements were obtained from 19 channels according to the International 10–20 System electrode positions. Considering the characteristics of the CI and the theta waves (θ) occurring in multiple regions, data from eight channels were analyzed: prefrontal Fp1, Fp2, frontal F3, F4, parietal P3, P4, and occipital O1 and O2 (highlighted in gray in Figure 1d).

The experimental preparation was as follows:

1. The day before the experiment, the participants were instructed not to consume any alcohol or caffeinated substances. On the day of the experiment, participants reviewed the experimental information and signed a consent form.
2. Before beginning the experiment, the experimental space was prepared (turning off the air conditioning system, drawing curtains, closing sliding doors, and adjusting the lights). Once the participant was seated in the experimental area, the researcher fitted the individual with EEG equipment and adjusted the fit for each participant.
3. With the participant wearing the EEG equipment, a brainwave diagnostic process for the EEG impedance check was conducted using the DSI-Streamer-v.1.08.44 program. Once the impedance of the attached 19 sensors dropped below 1, the experiment was initiated.
4. The experiment consisted of EEG recording and subjective response. The experiment was opened with Guide 1 (instructions to maintain a comfortable posture and minimize physical movement and introduction of next-screen content), followed by a 15 s gray image Ref1, Guide 2 (introduction of observation time for each stimulus and the gray screen between them, 30 s eye closure), a 30 s relaxation period for the participant with closed eyes, Guide 3 (instruction to open eyes and begin the experiment), and finally, experimental content sets A and B in a random order. Half the participants were presented with set A and the other half with set B. The sequence in content sets A and B included Ref2 (15 s gray image), the first 15 s stimulus, a 15 s gray image for afterimage prevention, the second 15 s stimulus, continuing in this manner and ending with 15 s of the 19th stimulus. The experimental duration was 10 min and 50 s per participant. Finally, Guide 4 (thank you message and introduction to move to the next table for the postsurvey questionnaire) was provided, and the EEG recording was completed.
5. After the experiment, the participants completed a self-report survey on demographic information and the selection of one room where they felt they would be the most focused and one where they felt they would be the most creative.

**Figure 2:**
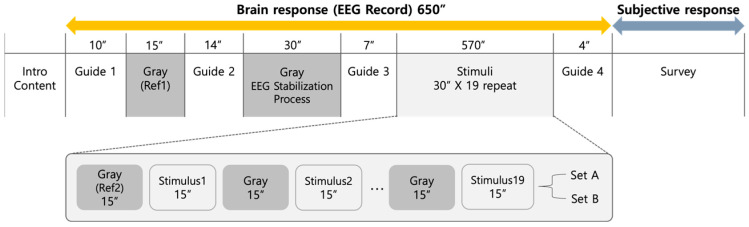
Experimental procedure.

Figure 2 represents the actual experiment procedures:

### 3.4. Measurement and Analysis of EEG Data

#### 3.4.1. Measure and Data Collection

The CI derives from (SMR + mβ)/θ, a combination of beta waves and SMR waves [51], which tended to manifest in the frontal and parietal lobes [46,48]. Hence, the index applied in this study encompasses various parts of the brain, and a comprehensive analysis of each area is necessary. Data were recorded by connecting a computer to the DSI-24 EEG system via a cable and using DSI-Streamer-v.1.08.44 software. Noise, including eye blinks and muscle movements, was filtered using TeleScan software (TeleScan. version 3.2; Laxtha, Seoul, Korea).

#### 3.4.2. EEG Data Indicators and Analysis

The raw data were transformed using a fast Fourier transform (FFT), and the Power Spectral Density (PSD) of the signal was calculated for each channel, with the unit expressed in microvolts squared per hertz μV^2^/ Hz [65]. In alignment with the objectives of this study, the total power within specific frequency and time ranges was selected and the Frequency Band Power extracted. This metric illustrates the level of brain activity within a particular frequency band. By analyzing the Frequency Band Power values, we can ascertain the distribution of signal power across various frequencies within the EEG signal.

To explore the statistical differences in brainwaves among stimuli and compare the EEG differences between different stimuli, this study used the Wilcoxon signed-rank test, which is also a commonly used method for EEG signal analysis [65,66]. The paired t-test is suitable for data from a large sample and requires the paired dataset to follow a normal distribution; however, when testing the normality of randomly sampled data from the data we collected for this study, most *p*-values were below 0.05, indicating that the collected data did not follow a normal distribution. Therefore, the nonparametric Wilcoxon signed-rank test, which is used to test for differences between paired samples, was used in this study. The Wilcoxon signed-rank test was used to determine whether there was a difference between “n” paired samples and was tested by ranking the observed scores of the two samples. The significance level was set at *p* < 0.05 and *p* < 0.01.

## 4. Results

### 4.1. Distribution of EEG by Stimulus and Channel

#### 4.1.1. Distribution by Stimulus

**Figure 3:**
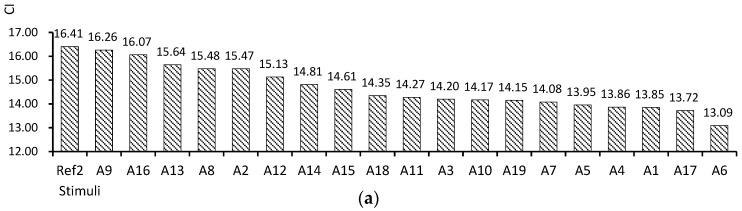

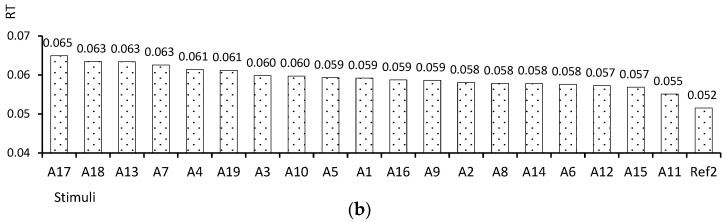
(**a**) CI average per stimulus; (**b**) RT average per stimulus.

The average EEG data of the 20 participants for each stimulus are shown in Figure 3. All CI values fell between 33.70 and 3.33, with the highest average value observed when participants viewed Ref2, the gray screen shown before the stimuli. When ranked in descending order of CI values, the stimuli with the highest CI were Ref2, A9, A16, A13, A8, A2, A12, A14, A15, and A18, with the lowest value for A6. All RT values fell between 0.13 and 0.01. The highest average value was observed for stimulus A17. The stimuli were ranked in descending order as A17, A18, A13, A7, A4, A19, A3, A10, and A5, with the lowest value for Ref2.

For the CI, looking at the characteristics of the top nine stimuli (half of the total), a relatively high prevalence of rectilinear, traditional furniture arrangements, low ceilings, and red were observed. In the case of the RT, no common features appeared in terms of shape, furniture arrangement, or ceiling height; however, blue color was frequently observed. Additionally, the top-ranked color was white (A17), and the second-ranked stimulus featured a wall made entirely of glass (A18), revealing natural scenery. Thus, when averaging EEG brainwave data across eight channels, brainwave indicators related to concentration tended to be activated by rectilinear forms, traditional arrangements, low ceilings, and red. Conversely, brainwave indicators related to creativity were activated by white, blue, and a view of the natural environment.

#### 4.1.2. Distribution by Channel

**Figure 4:**
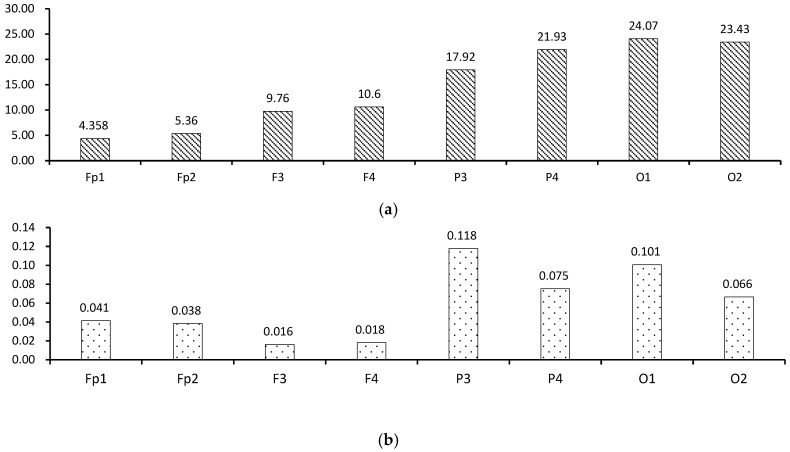
(**a**) CI average per channel; (**b**) RT average per channel.

The average EEG data of the 20 participants for CI and RT for each channel are summarized in Figure 4.

Overall, the participants’ brainwaves in response to the stimuli in this study were observed more frequently in the parietal (P3, P4) and occipital lobes (O1, O2) than in the prefrontal cortex or frontal lobe.

The highest CI values were observed in occipital lobes (O1 and O2), followed by P, F, and Fp. When the left and right hemispheres were compared, the values of Fp, F, and P were slightly higher on the right than on the left. This conclusion is consistent with the view proposed by Çiçek et al. [67] and Okon-Singer et al. [68] that the right brain plays the leading role in attention allocation.

For RT, the highest values were primarily located in the parietal lobe P, followed by O, Fp, and F. Comparing the left and right hemispheres, the values in Fp, P, and O were higher on the left side than on the right. In theta waves, Chowdhuri and Mal‘s study [69] also found a similar conclusion that the PSD value of the left hemisphere theta wave is higher than that of the right hemisphere. Thus, CI and RT values showed opposite tendencies in the left and right hemispheres.

### 4.2. Differences in EEG Response Before and After Stimuli of Indoor Environmental Elements

To gain a more detailed understanding of the impact of stimuli on participants’ brainwaves, the Wilcoxon test was used to examine the statistical differences in brainwave channels before and after each stimulus. The gray background stimulus Ref2 and 19 other stimuli formed pairs without duplication or change in order, resulting in 190 combinations (C (20,2) = $\frac{20!}{2!\left(20-2\right)!}$). With eight brainwave channels for each combination, 1520 statistical results (8 × 190) were obtained for each brainwave indicator.

The differences between the grey screen (Ref2) shown before the stimuli and the stimuli themselves were investigated using the Wilcoxon signed-rank test. The data averaged during the 15 s of viewing the gray screen (Ref2) was used as the pre-stimulus condition. The grey screen, which was neutral in color and devoid of any other stimuli, served as a reference point for brainwave activity without stimulation. Table 3 shows the statistically significant results.

In the CI group, the stimuli that showed a significant difference from Ref2 were A1, A2, A3, A4, A5, A6, A7, A8, A11, A12, A14, A15, A16, A17, A18, and A19. Differences were primarily observed in the O1 channel of the left brain, followed by the O2 and F4 channels. For these stimuli, the CI was significantly lower in some channels in most stimuli than in Ref2, suggesting a decrease in concentration after the stimulus compared to the pre-stimulus condition.

For the RT, the stimuli that showed a significant difference from Ref2 were A1, A2, A3, A4, A5, A6, A7, A8, A13, A14, A15, A17, A18, and A19. Significant differences were observed between the O1 and O2 areas. Among these stimuli, RT was significantly higher in some channels of most stimuli than in Ref2, suggesting an increase in creativity after the stimulus compared to the pre-stimulus condition. Overall, significant differences between Ref2 and the stimuli in CI and RT were most frequently observed in occipital lobes O1 and O2.

### 4.3. Differences in EEG Response by Indoor Environmental Elements

A Wilcoxon test was conducted to examine whether brainwave responses were significantly different when only one indoor environmental element differed and the other three elements were identical (see Table 4).

The results showed significant differences in the CI of the F3 and P4 channels across the three cases. Under the conditions of the traditional arrangement, low ceiling height, and red color, higher CI values were observed in rectilinear forms (A16) compared to curvilinear forms (A1). Higher CI values were also observed in the traditional furniture arrangement (A15) under conditions of rectilinear forms, high ceiling height, and red color, compared to the collaborative furniture arrangement (A10). Additionally, under the conditions of curvilinear forms, collaborative arrangement, and low ceiling height, red (A2) showed a higher CI than blue (A6).

For the RT, significant differences were found in channels Fp1, Fp2, F4, P3, P4, O1, and O2 across the four design elements. Higher RT values were found in curvilinear forms (A1, A2, A3, and A4) and collaborative arrangements of furniture layouts (A10 and A11). There was a lack of consistency in ceiling height and color. Overall, rectilinear forms, traditional furniture arrangements, and red furniture tended to have higher CI, whereas curvilinear forms and collaborative furniture layouts showed higher RT.

When examining the brain areas, we found that significant differences in CI: F3 were prominent. For the RT, Fp2 and P4 were the most prominent markers.

### 4.4. Subjective Response Analysis

The participants’ subjective responses to the survey questionnaire were analyzed in terms of frequency. In response to the question about the stimuli in which they felt they could best concentrate, they selected the classroom with the white wall most often (S17, 45%), followed by S12 (20%), S7 and S8 (10% each), and S5, S16, and S19 (5% each). A common feature is a traditional front-facing linear furniture layout. For creative thinking, participants selected S18 (full nature view, 45%), followed by S4 (25%), S11 and S3 (10% each), and S1 and S6 (5% each). The common features were the collaborative furniture layout and curvilinear shape. (See Table 5).

### 4.5. Comparison of EEG Differences by of Stimuli with A17 (White Walls) and A18 (View of Nature)

Because participants selected A17 as best for concentration, a Wilcoxon test was conducted to analyze whether statistically significant differences occurred between A17 and other identical stimuli in all aspects, except for wall configurations (A13 with blue walls, A16 with red walls, and A18 with natural scenery). A17 featured characteristics commonly seen in typical school classrooms, including rectilinear forms, traditional frontal furniture arrangements, low ceiling heights, and white walls. Table 6 presents the statistically significant results.

The results showed that in terms of CI, stimuli with significant differences from A17 were A13 with blue walls and A16 with red walls. The channels that showed significant differences were O1, O2, and F4. Higher CI values were observed for these stimuli than for A17, with the most significant differences observed in A16, which had red walls. This suggests that spaces with red or blue walls tended to have higher CI values than those with white walls. This EEG result contrasts with that of the subjective response because A17 was selected as the best space for concentration. Regarding RT, A13 (blue) and A16 (red), with differences observed in channels O1, F4, and P4, exhibited lower RT values than A17. In A16 with red walls, the most substantial differences were observed across the three channels; therefore, it can be inferred that white walls produced the highest RT values, followed by blue and red walls.

Because participants selected A18 as best for creativity, a Wilcoxon test was conducted to analyze whether statistically significant differences occurred between A18 and other stimuli that were identical in all aspects, except for wall configuration. Stimulus A18 featured rectilinear forms, a traditional frontal table layout, low ceilings, and walls made entirely of glass, revealing natural scenery. The results showed that the RT was significantly higher in A18 than in A16. This suggests that spaces with views of natural scenery tend to have higher creativity than those with red walls. Meanwhile, CI was significantly higher in A16 than in A18. This suggests that spaces with red walls tend to have higher concentration but lower creativity than those with views of natural scenery. In other words, creativity tended to be higher in environments with natural views than in those with red walls.

### 4.6. Comprehensive Characteristics of EEG Response and Subjective Response

Table 7 summarizes the comprehensive results of the brainwave response analyses and subjective response. The findings indicate that for CI related to concentration, the closely associated features were red, rectilinear shapes, and traditional furniture arrangements. In contrast, for RT related to creativity, the closely associated features were curvilinear shapes, collaborative furniture arrangements, and walls made entirely of glass, revealing views of natural scenery. The subjective response shows that participants selected white walls and a front-facing furniture layout as supportive of concentration and a full view of nature, curvilinear shape, and collaborative furniture layout for creative thinking (See Table 7).

## 5. Discussion

### 5.1. Characteristics of Indoor Environmental Elements Related to Concentration and Creativity

In this study, the characteristics of the indoor environmental elements related to concentration included rectilinear forms, traditional furniture arrangements, and red. The results of this study on traditional frontal furniture arrangements related to concentration align with research suggesting that traditional furniture arrangements help students maintain their attention on the teacher [32]. The finding that red enhanced attention more than the reference stimulus of white supports previous research indicating that warm colors (yellow, red) enhanced attention more than cool colors [9] and that red was more effective in improving attention to detail [36]. Warm-colored walls, such as yellow and red, have shown to improve attention and enhance learning outcomes [9]; however, contrasting reports have suggested that dark red led to the lowest concentration [19]. This might have occurred because the various luminance, hue, and saturation levels in the studies resulted in differing emotions and responses.

The characteristics of the interior design elements related to creativity identified in this study include curvilinear forms, collaborative furniture arrangements, white, and walls made entirely of glass, revealing natural scenery. The findings align with the results of prior studies, suggesting that a rounded physical work environment has a higher potential to induce creativity [29]. These findings were also inferred from studies linking the emotional states of participants with enjoyment and pleasure derived from curvilinear forms [17,28]. In addition, the finding that a collaborative furniture arrangement is related to creativity is consistent with other studies showing that clustered furniture layouts enhance student interaction and creativity [32] and that flexible and diverse furniture arrangements foster creativity [34].

Our finding of the positive impact of natural elements on creativity is also consistent with prior studies showing (a) more creativity in product design work in natural environments [44], (b) enhancement of higher-level tasks such as creative problem-solving when immersed in nature [70], and (c) creative actions and creative thinking induced by multisensory stimulation from natural environments [45]. Being in an outdoor natural setting or simply viewing nature indoors is sufficient to stimulate creativity [71], indicating a close relationship between natural elements and creativity. In terms of colors related to creativity, this study showed that white was more strongly associated with creativity than red or blue. This finding is not in line with prior studies that have indicated that cool colors stimulate creative potential [39], blue and red accent lighting indirectly improved creative performance [40] and green enhanced creativity [72]. A possible reason for this difference may relate to various indicators used in each study. For example, in current study we used the RT of EEG as the indicator for creativity, but other studies used survey results from 60 managers’ rating of the creativity potential [31], and other survey results [40].

### 5.2. EEG Activation Characteristics Related to Concentration and Creativity

Our examination of brainwave results showed that CI, representing concentration, and RT, representing creativity, exhibited somewhat opposite tendencies. For example, rectilinear shapes tend to have a higher CI, whereas curvilinear shapes yield a higher RT. Similarly, traditional furniture arrangements were correlated with higher CI, whereas collaborative arrangements were associated with higher RT. Prior research has indicated that creativity involves diffused attention, a brain activity that is somewhat contrary to that of focused attention [73]. Neuroimaging studies have shown that attention and working memory share “a functional overlap in the mechanisms” [73] (p. 125) and neural substrates. Conversely, other fMRI studies examining the relationship between creativity and working memory found that creativity, as measured by divergent thinking tests, was associated with an inefficient reallocation of concentration, linking creativity with diffuse attention. These findings allow us to infer that concentration and creativity have contrasting characteristics; thus, indoor environments optimized for concentration and creativity will likely have some degree of contrasting characteristics.

Red is known to evoke attention [74]; it often signifies an alert. In traditional rectilinear furniture arrangements, the environment is relatively tense, which can increase the CI. Contrarily, curvilinear space elements are less common in classroom environments and can provide feelings of uniqueness and originality relevant to creativity. Collaborative furniture arrangements are associated more with a gentle atmosphere conducive to teamwork and communication than attention focused on the lecturers. White and natural scenery seemed to increase RT values, avoiding strong color stimuli that demand attention.

### 5.3. Brainwave Activation Area

In the present study, CI was higher in the occipital and parietal lobes, and RT was higher in the parietal and occipital lobes. The higher observation in the occipital lobe was likely the result of strong visual stimuli inducing brainwaves [48,55]. The parietal lobe integrates information related to motor–spatial attention and visual–spatial memory [75].

Overall, the RT indicator tended to be higher in the left side of the brain than in the right side. This aligns with a prior study showing that, in healthy individuals, the left frontal lobe can inhibit the right hemisphere in specific and creative thinking [76]. When examining whether the brainwave responses were significantly different when only one indoor environmental element differed, we found that the brain locations showing significant differences were F3 (CI), FP2, and P4 (RT), which were mostly skewed toward the front of the brain. In particular, for stimuli in which furniture, ceiling height, and color varied, significant differences were frequently observed in the frontal and prefrontal lobes. This could be related to the association of frontal areas of the brain with higher cognitive function, thought, and attention [49].

### 5.4. Comparison of Brain Response with Subjective Response

The subjective responses show some similarities and differences with brain response. Regarding similarities, subjective responses to front-facing furniture for concentration aligned with high CI for front-facing furniture. Regarding creativity, the participants perceived the best creative thinking in a classroom with a full view of nature, which coincided with a high RT. Additionally, the subjective response to creativity with collaborative furniture aligned with a high RT. Of the four indoor environmental elements, furniture layout seemed to be most closely related to their perception of concentration and creativity. Regarding differences, the subjective response revealed that the white wall was perceived as the best for concentration, but the brain response showed a high CI for the red wall and lowest CI for while wall. Compared to related studies, Liu [9] found that more concentration-related beta waves were generated by warm colors (yellow and red) than by white, and participants reported that they felt they would perform better when surrounded by warm colors, suggesting that the EEG responses resembled the findings of this study, but the subjective responses differed.

Several prior studies also show differences between EEG and subjective response. For example, in a study of the influence of the shape of architectural space on human brain activity and emotional responses, Banaei et al. (2017) observed discrepancy between the two, explaining that the difference might result from the gaps between real-time EEG data and the post evaluation of emotion (measured by the Self-Assessed Manikin [SAM] test) [17]. In other words, EEG shows the immediate influence of shape on dynamic changes of the brain while the SAM test allows the evaluation of emotional impact after fully experiencing the entire room. In another study, Mavros et al. (2022) also observed discrepancy and explained that it may result from the difference in the immediate response of EEG and the long-term recognition of emotional responses [77]. To illustrate, in a crowded environment, negative self-reported valence was observed; but the EEG responses showed positive responses with frontal alpha asymmetry, suggesting that although people reported feeling uncomfortable in crowded spaces, the EEG data suggest that they may show positive neurophysiological responses in such situations. Cho et al. (2023) reported discrepancy between the psychological selection and relative alpha to beta metric of EEG on the healing environment [78].

The other possible reason for discrepancy may depend on the psychological construct being investigated, highlighting the complexity of brain–behavior relationships in naturalistic settings. Both self-reported emotional response and EEG provide meaningful data to facilitate the understanding of responses from different perspectives, with EEG being able to “convey information about the user state without directly intruding into the user’s consciousness or task at hand” [79], allowing for a more multidimensional and comprehensive interpretation of the human response to environment and fully understanding of human perceptual evaluation of environments [80].

### 5.5. Suggestions for Design Applicaiton

In a world where smart technology and artificial intelligence are increasingly used to support remote work and learning and where the spatial boundaries between home, the learning environment, and the workplace are blurred, identifying environmental characteristics that facilitate concentration and creativity is significant for classroom environments and many other spatial designs. As concentration and creativity are understood not to be fixed but malleable and nurtured, the potential to enhance them through interior design is significant.

First, the use of color and furniture arrangements is an affordable recommendation for achieving the desired learning effects: Furniture arrangements can be modified with relative ease owing to their mobility and flexibility. Color is relatively easy to alter without changing the spatial structure. Second, to enhance creativity, a curvilinear form may be considered when designing or selecting walls, lighting fixtures, furniture shapes, or other classroom objects. Third, because common results from EEG and subjective responses show that frontal furniture arrangement encourages concentration and collaborative arrangement, curved shapes, and natural views encourage creativity, we suggest that these elements be prioritized in designing educational spaces. Incorporating biophilic design into breakout areas inside and outside classrooms can encourage creativity and recreation. In the overall planning of a school or educational space, we recommend organizing spaces with different characteristics so that each space can be selected and used according to the educational goals.

## 6. Conclusions

### 6.1. Summary

Using EEGs in this study, we explored how various elements of space—space shape, furniture arrangement, ceiling height, and color—along with images of white walls, a full wall of windows with a view of nature, and a windowless scenario impact brainwaves, particularly in terms of concentration and creativity indicators, in addition to subjective responses.

The research conclusions can be summarized as follows:

1. Our review of the literature shows that some researchers have used EEG responses to examine the effects of indoor environments on concentration, but few have examined the effects on creativity. In addition, some have examined the effects of color, furniture arrangement, and biophilia on concentration or creativity, but few have examined the effects of ceiling height and shape. This review indicates a strong need for more studies to be conducted in the context of the classroom environment using EEG.
2. The CI was highest in Ref2 (gray reference image), whereas the RT was highest in A17 with a white wall. Before stimulation has high value in CI while after stimulation has high value in RT. The brainwaves related to high CI were primarily located in the occipital lobe and parietal lobe, followed by the frontal lobe and prefrontal lobe. Brainwave data with high RT were primarily on the left side in the following order: P, O, Fp, and F.
3. Statistical analysis of EEG responses showed that the features most related to CI were rectilinear forms, traditional furniture layout, and red; the features most related to RT were curvilinear shapes, collaborative layout, white, and walls made entirely of glass, revealing natural scenery.
4. The subjective responses showed that a classroom with white walls was perceived as the best for concentration and that with a full view of nature was the best for creative thinking. Participants perceived the traditional furniture layout as better for concentration, and the collaborative layout and curvilinear shape for creative thinking.
5. In a comparison of brain and subjective response, responses to front-facing furniture layout were associated with concentration, while a view of nature, collaborative furniture layout, and curvilinear shape were associated with creativity aligned. Responses to color, however, differed.
6. The similarities between brain and subjective responses may provide more robust evidence, but the differences between them need to be better understood. These could be individual differences or factors related to subjective preferences. Looking at both brain and subjective responses together is worthwhile for more comprehensive evidence-based design and research.

### 6.2. Limitations and Future Research Directions

The limitations of this study are as follows. First, because of the practical challenges in altering real environments, we used rendered images, which may differ from the experience in actual spaces. To minimize the influence of external factors, the laboratory was designed with plain white walls and no decorations. Although using a virtual reality head-mounted display can block the surroundings, concerns about discomfort and potential brainwave interference from wearing both the VR and EEG equipment led to the decision to conduct the experiment using a monitor. Second, the small number of participants (N = 20) made generalization of the results difficult. Further research with larger sample sizes is necessary to derive additional generalizable conclusions. Third, as we measured brain and subjective responses but not specific tasks involving concentration and creativity, it is uncertain whether people actually perform better in classrooms with revealed stimuli characteristics. Based on the predominant influencing characteristics found in this study, we will reduce the number of stimuli in our next study and compare EEG, subjective, and task performance. In this study, EEG was measured in the absence of a task, but the similarity of EEG and subjective responses to several indoor elements suggests that these elements are more potentially powerful influences. Fourth, this study examined subjective responses by asking participants to choose the classroom where they perceive most supportive for concentration or creativity, but scale ratings were not available for all stimuli, so within-subject correlation analysis could not be conducted.

Thus, we recommend that future researchers use tasks or tools to measure concentration and creativity, and compare brainwave indicators with task performance as well as more detailed subjective responses for a more comprehensive and holistic understanding. Additionally, the use of lightweight EEG equipment with VR headsets may be beneficial. Studies have shown that the cognitive EEG results and task outcomes are similar in virtual and real environments [62], suggesting the usefulness of VR in controlling stimuli and observing brainwave changes. However, EEGs have limitations because they only measure surface brain activity and do not show deeper brain activity. The amygdala in the brain is known to be related to consciousness and creativity [81]; therefore, exploring subsurface brainwave changes with higher spatial resolution techniques, such as fMRI, seems worthwhile for a comprehensive understanding. In addition, to ensure the reproducibility of the study, researchers must report their methods in detail to contribute to the establishment of a more comprehensive way of understanding the effects of the environment on humans.

We believe that this study contributes to the community of environmental design and research because it (a) examines EEG responses relevant to concentration and creativity, (b) contributes to the understanding of the characteristics of interior design that appear to enhance the manifestations of concentration and creativity, and (c) broadens our understanding of the similarities and differences between brain responses and subjective responses.

## Figures and Tables

**Table 1:** Summary of EEG studies on classroom design.

Authors	Variables	Participant #	EEG Indicator	Findings
Zhang et al. (2024) [18]	4 classroom size (a) SHR: 9.0 m × 4.5 m × 3.9 m; (b) SLR: 9.0 m × 4.5 m × 3.0 m; (c) BLR: 9.0 m × 6.9 m × 3.9 m; (d) BHR: 9.0 m × 6.9 m × 4.8 m	34	Beta	Higher subjective ratings occurred in larger classrooms with the same ceiling height. The task test improved by 17.3% in the BHR and by 20.1% in the SLR. Physiological data revealed significant effects of ceiling height, with EEG-β power increasing by 53.9% in BHR and by 22.8% in SLR.
Llinares et al. (2021) [8]	12 warm and 12 cold hue color settings in a virtual classroom	160	Beta High-β	Cold hue colors increase arousal and improve performance in attention (respond to sounds) and memory tasks.
Liu et al. (2022) [9]	5 virtual classrooms with yellow, red, white, blue, and green walls Stroop task, a digital calculation, and reading	34	Low-β High-β	Survey results: The cold-colored walls (blue and green) had the highest levels of relaxation and pleasure while the warm-colored walls (yellow and red) supported better attention and learning performance. The white walls had the lowest subjective evaluation and the worst learning performance. The yellow wall had the highest low-β and high-β in the FP2 channel.
Kim and Gero (2022) [55]	7 different biophilic classroom design cases	17(13)	Relative alpha, beta	High relative beta in the parietal and occipital lobes of students in classrooms with biophilic elements.
Zhang, Y. et al. (2024) [56]	Natural window views and building window views	30	Theta, alpha, and beta	Natural window views provided a sense of comfort, relaxation, and pleasure and increased learning efficiency compared to the building window view.
AE Nieto-Vallejo et al. (2021) [57]	Light color temperature in 2500 K to 6500 K	14	14 electrodes	Warm colors were more useful for concentration when performing multiple activities (listening, writing, observing) as opposed to cooler colors for single tasks.
Fu et al. (2023) [58]	3300 K/300, 500, 750 lx 4300 K/300, 500, 750 lx 5300 K/300, 500, 750 lx	46	Alpha	CCT of 3300 K and illuminance of 300 lx was more comfortable than other combined conditions.
Fu, X. et al. (2023) [59]	3300 K, 4300 K, and 5300 K 300 lx, 500 lx, and 750 lx	24	Alpha	The attention level tended to increase linearly; the attention level was the lowest at 300 lx and the highest at 500 lx.
Llinares et al. (2021) [60]	3 classroom width settings (8.80, 8.20, and 7.60 m)	90	C3-Beta CZ-High beta	Wider classrooms are associated with poorer performance and lower emotional arousal.
Juan and Chen (2022) [61]	Lighting, sound, temperature, and smell	68	Beta	Temperature has the greatest impact on concentration.
Kalantari et al. (2021) [62]	Virtual reality space and real space with the same layout	25	Delta, theta, alpha, beta, gamma	Concentration levels were similar in virtual and real environments.

**Table 2:** Elements in experimental stimulus and 19 stimuli.

Code	Shape		Furniture Arrangement		Ceiling Height		Color
1	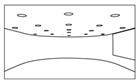		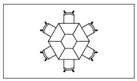		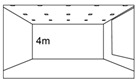		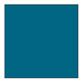
	Curvilinear		Collaborative		High (4 m)		deep blue 8.7B 4/5
0	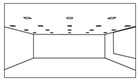		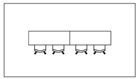		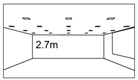		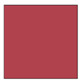
	Rectilinear		Traditional		Low (2.7 m)		deep red2.5R 4/8
A1	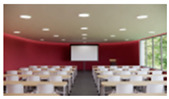	A2	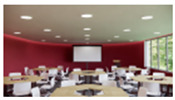	A3	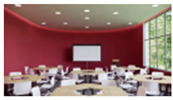	A4	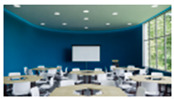
	S1/FA0/CH0/C0		S1/FA1/CH0/C0		S1/FA1/CH1/C0		S1/FA1/CH1/C1
A5	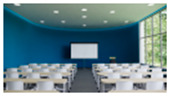	A6	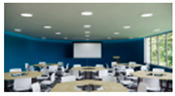	A7	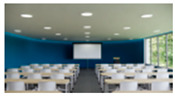	A8	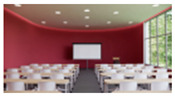
	S1/FA0/CH1/C1		S1/FA1/CH0/C1		S1/FA0/CH0/C1		S1/FA0/CH1/C0
A9	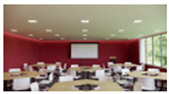	A10	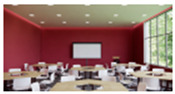	A11	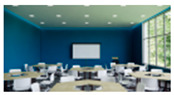	A12	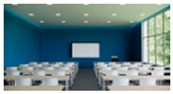
	S0/FA1/CH0/C0		S0/FA1/CH1/C0		S0/FA1/CH1/C1		S0/FA0/CH1/C1
A13	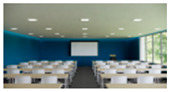	A14	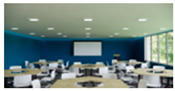	A15	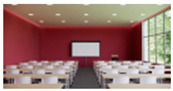	A16	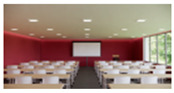
	S0/FA0/CH0/C1		S0/FA1/CH0/C1		S0/FA0/CH1/C0		S0/FA0/CH0/C0
A17	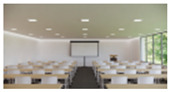	A18	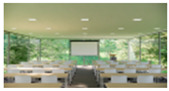	A19	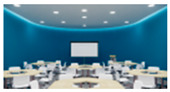	REF	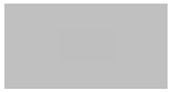
	S0/FA0/CH0/White		S0/FA0/CH0/Window		S1/FA1/CH1/C1No-Window		-

Notes: “Shape” refers to the shape of the space, the shape of the ceiling lighting, ceiling lighting arrangement, and corners of the tables. Each space was designed with 48 seats. S (Shape): 1 = Curvilinear (CL), 0 = Rectilinear (RL). FA (Furniture Arrangement): 1 = Collaborative (C), 0 = Traditional (T). CH (Ceiling Height): 1 = High (H), 0 = Low (L). C (Color): 1 = Blue (B), 0 = Red (R).

**Table 3:** Difference between the stimulus and the reference value.

Comparison		CH1 (Fp1)	CH2 (Fp2)	CH4 (F3)	CH5 (F4)	CH16 (P3)	CH17 (P4)	CH18 (O1)	CH19 (O2)	Count
CI	**Ref2**-A1		2.34 *		−2.18 *			−3.19 **		3
	**Ref2**-A2							−2.51 *	−2.30 *	2
	**Ref2**-A3								−2.34 *	1
	**Ref2**-A4							−3.05 **		1
	**Ref2**-A5							−3.94 **		1
	**Ref2**-A6							−2.91 **		1
	**Ref2**-A7				−2.05 *			−2.09 *		2
	**Ref2**-A8				−2.38 *					1
	**Ref2**-A11							−2.91 **		1
	**Ref2**-A12							−2.68 **		1
	**Ref2**-A14							−3.19 **		1
	**Ref2**-A15							−2.14 *		1
	**Ref2**-A16							−2.14 *		1
	**Ref2**-A17							−3.00 **	−2.38 *	2
	**Ref2**-A18				−2.26 *			−2.14 *	−1.97 *	3
	**Ref2**-A19							−2.51 *		1
Count		0	1	0	4	0	0	14	4	
RT	Ref2-**A1**				1.00 *			1.00 *		2
	Ref2-**A2**							0.67 **	0.47 **	2
	Ref2-**A3**							0.81 *	0.93 *	2
	Ref2-**A4**							0.59 **	0.65 **	2
	Ref2-**A5**							0.61 **		1
	Ref2-**A6**							1.02 *		1
	Ref2-**A7**							1.00 *		1
	Ref2-**A8**							0.90 *		1
	Ref2-**A13**	0.81 *						0.94 *	1.00 *	3
	Ref2-**A14**				0.63 **			0.65 **		2
	Ref2-**A15**							1.00 *		1
	Ref2-**A17**							0.59 **	0.92 *	2
	Ref2-**A18**								0.71 **	1
	Ref2-**A19**							1.02 *	0.77 *	2
Count		1	0	0	2	0	0	13	7	

Note: Z value *: *p* < 0.05, **: *p* < 0.01; bold text means that stimulus has larger value.

**Table 4:** Differences between single design elements.

Comparison			CH1 (Fp1)	CH2 (Fp2)	CH4 (F3)	CH5 (F4)	CH16 (P3)	CH17 (P4)	CH18 (O1)	CH19 (O2)	Count
CI	Shape	A1-**A16**						−2.34 *			1
	Furniture layout	A10-**A15**			−2.05 *						1
	Ceiling height	/									0
	Color	A6-**A2**			−2.14 *						1
Count			0	0	2	0	0	1	0	0	
RT	Shape	**A1**-A16						0.96*	1.02 *		2
		**A2**-A9							0.96 *		1
		**A3**-A10								0.90 *	1
		**A4**-A11					0.94 *				1
		A6-**A14**				1.02 *					1
	Furniture layout	**A10**-A15	1.02 *								1
		**A11**-A12		0.81 *							1
	Ceiling height	A12-**A13**	0.82 *	1.00 *							2
		**A15**-A16						0.96 *			1
	Color	A12-**A15**		0.67 **							1
		**A13**-A16						1.02 *			1
Count			2	3	0	1	1	3	2	1	

Note: Z value *: *p* < 0.05, **: *p* < 0.01; bold text means that stimulus has larger value.

**Table 5:** Order and stimulus characteristics.

Concentration						Creative Thinking					
Stimulus	Frequency	S	FA	CH	C	Stimulus	Frequency	S	FA	CH	C
A17	9 (45%)	0	0	0	White	A18	9 (45%)	0	0	0	Window
A12	4 (20%)	0	0	1	1	A4	4 (25%)	1	1	1	1
A7	2 (10%)	1	0	0	1	A11	2 (10%)	0	1	1	1
A8	2 (10%)	1	0	1	0	A3	2 (10%)	1	1	1	0
A5	1 (5%)	1	0	1	1	A1	1 (5%)	1	0	0	0
A16	1 (5%)	0	0	0	0	A6	1 (5%)	1	1	0	1
A19	1 (5%)	1	1	1	1 no-window						

Note: S (shape): 1 = curvilinear (CL), 0 = rectilinear (RL); FA (furniture arrangement): 1 = collaboration (C), 0 = traditional (T); CH (ceiling height): 1 = high (H), 0 = low (L); C (color): 1 = blue (B), 0 = red (R).

**Table 6:** Differences between stimulus and standard stimuli A17 and A18.

Comparison			CH1 (Fp)	CH2 (Fp2)	CH4 (F3)	CH5 (F4)	CH16 (P3)	CH17 (P4)	CH18 (O1)	CH19 (O2)	Count
CI	**A13**-A17								−2.47 *	−2.05 *	2
	**A16**-A17					−1.97 *			−2.22 *	−2.82 **	3
Count			0	0	0	1	0	0	2	2	
RT	A13-**A17**								0.81 *		1
	A16-**A17**					0.65 **		0.41 **	0.94 *		3
Count			0	0	0	1	0	1	2	0	
CI		**A16**-A18				−2.01 *				−2.18 *	2
Count			0	0	0	1	0	0	0	1	
RT		A16-**A18**						1.00 *			1
Count			0	0	0	0	0	1	0	0	0

Note: Z value *: *p* < 0.05, **: *p* < 0.01; bold text means that stimulus has larger value.

**Table 7:** Main findings about brainwave responses and subjective responses related to concentration and creativity.

Responses	Elements	Concentration						Creativity					
		Shape	Furniture Arrangement	Ceiling Height	Color	A17 (White wall)	A18 (View of nature)	Shape	Furniture Arrangement	Ceiling Height	Color	A17 (White wall)	A18 (View of nature)
Brain response	High index in EEG (CI in concentration, RT in creativity each)	Rectilinear	Traditional arrangements	-	Red	Red > White Blue > White	Red > natural scenery	Curvilinear forms	Collaborative arrangements	-	-	Red < White Blue < White	Red < natural scenery
	Channel	P4	F3	-	F3	F4, O1, O2 O1, O2		P3, P4, O1, O2	Fp1, Fp2	-	-		P4
Subjective response	High in frequency		Traditional arrangements			White walls		Curvilinear	Collaborative arrangements				Natural scenery

## Data Availability

The data presented in this study are available on request from the corresponding author. The data are not publicly available due to privacy.
